# Profiles in neglect of older adult care workers in a long-term care facility: a latent profile analysis

**DOI:** 10.3389/fpubh.2024.1320896

**Published:** 2024-03-25

**Authors:** Chang Yan, Caili Wang, Xinshu Ding, Lefei Sun, Wei Gao, Deshan Liu

**Affiliations:** ^1^School of Nursing and Rehabilitation, Shandong University, Jinan, China; ^2^PICC Clinic, Qilu Hospital of Shandong University, Jinan, China; ^3^Department of Traditional Chinese Medicine, Qilu Hospital of Shandong University, Jinan, China

**Keywords:** older adult care workers, older adult abuse, older adult mistreatment, latent profiles analysis, long-term care facilities (LTCFs)

## Abstract

**Background:**

Neglect is a common form of abuse, and long-term care facilities record higher incidences of this abuse. Given that older adult care workers are the main workforce in these facilities, their neglectful behavior requires public health attention. Internal individual characteristics can lead to older adult abuse, and managing workers who abuse older adults may require various methods. This study aimed to identify the profiles of neglect among older adult care workers in long-term care facilities and explore the influencing factors of neglect.

**Methods:**

In this cross-sectional study, a convenience sample of older adult care workers from 15 long-term care facilities in Shandong Province (*N* = 421) completed a questionnaire on the characteristics associated with neglect. Latent profile analysis was used to identify distinct neglect profiles and promote the understanding of individual characteristics associated with varying levels of neglect. One-way analysis of variance and multivariate logistic regression analyses were used to examine the population characteristic differences.

**Results:**

Older adult care workers exhibited three neglect profiles, namely, the “low-risk group,” “medium-risk group,” and “high-risk group.” Males, participants with no employment qualification certificate, and those who did not attend regular training represented the majority of those in the “high-risk group.” Participants with a monthly income of more than ¥ 4,000 and nursing 1–2 older adults simultaneously represented the majority of those in the “low-risk group.”

**Conclusion:**

Long-term care facility administrators should tailor interventions to individual care worker profiles to reduce neglect behaviors and improve care levels.

## Introduction

Population aging is a major developing global issue, including in China. The increase in aged population has introduced a significant burden on family care, which plays an important role in caring for older adults. At the same time, the transformation of the family structure, caused by the implementation of the family planning policy, development of the social economy, reduced fertility and mortality rates, and extended average life expectancy of the population, is bound to have a huge impact on the traditional home care model (1). China's family structure is gradually showing a trend of miniaturization and aging, and the proportion of “421” families, which means a couple needs to support four older adults and raise one child, is increasing yearly (2). Therefore, the number of family members available to take care of older adults is relatively reduced, which will eventually weaken the home care function and increase the burden and pressure on younger adults. Consequently, older adults gradually have reduced access to care resources provided by their families. It may be advantageous to focus on institutional care rather than on home care, and institutional care may become a main option for older adults (3).

The care for older adults in the majority of the institutions is provided by care workers. However, the high proportion of older adults and the shortage of care workers has increased the complexity of older adult care. The lack of nursing skills and improper attitude or behavior could lead to older adult abuse in the provision of older adult care (4). A 2020 meta-analysis showed that the incidence of older adult abuse in China was as high as 20.29% (5) while another study showed that a quarter of frail older adults were at risk of abuse and neglect, but only a small proportion could be identified (6). It is highly possible that such abuse is mostly ignored due to the lack of awareness of abuse among care workers. This may lead to serious consequences such as decreased quality of life, adverse health outcomes, and increased morbidity among older adults. The World Health Organization defines older adult abuse as “a single or repeated act or lack of appropriate action, occurring within any relationship where there is an expectation of trust, which causes harm or distress to an older person” (7). The five types of older adult abuse are, namely, physical abuse, mental abuse, economic abuse, sexual abuse, and neglect, with neglect being the most prevalent. Neglect refers to a relationship where an individual intentionally or unintentionally refuses to provide care to an older adult, cannot provide the older adult with the needed resources or services, or fails to protect the older adult from unnecessary harm. It can be divided into four types, namely, physical, psychological, economic, and medical neglect (8). In long-term care facilities, physical neglect is defined as the refusal or failure to provide basic goods or services to the older adult, including failure to provide adequate food, water, and clothing; aids; and safety protection. Physical neglect may lead to malnutrition, fecal impaction, stress ulcers, poor personal hygiene, or failure to seek medical care in older adults. Psychological neglect refers to the failure to ensure that older adults maintain normal social interactions, such as failing to provide any form of companionship for the older adult. Psychological neglect in older adults may lead to withdrawal, depression or anxiety, and ambivalence toward care workers. Economic neglect is the failure to utilize available funds and resources to maintain or restore the health of older adults, which is the case in situations where older adults are unaware of their financial situation or the sudden transfer of property to family members. Medical neglect refers to situations where older adults have unmet medical needs resulting from failure to take medications on time or attend medical check-ups. Studies have highlighted that the incidence of neglect in long-term care facilities is higher, ranging from 20% to 30% (9). Neglect may adversely affect a person's physical and mental health, causing temporary or long-term physical problems, anxiety, stress, sleep difficulties, and even suicidal ideations, and it may increase the risk of hospitalization and premature death (10). Therefore, neglect of older adults in long-term care facilities deserves public health attention.

Older adult care workers play a very important part in routine care for older adults, so they are the main source of neglect. The trait theory suggests that individual personality traits have a significant impact on their work behavior, and different behavioral tendencies trigger different behaviors. It is important to consider the personal characteristics and professional skills of older adult care workers, as they play a crucial role in older adult care and neglect. Previous research has focused on investigating the relationship between neglect and various factors such as work stress (11), social support (12), care setting (13), and professional attitudes (14). However, these studies have primarily examined correlations between variables, often using comprehensive scores to represent the overall situation without delving into individual differences in neglect.

Latent profile analysis (LPA) is a human-centered algorithm that can categorize responses from individuals with similar characteristics in different items and different categories with significant characteristics, ensuring the greatest difference between categories and the smallest difference within categories (15). In LPA, observed variable covariances are decomposed to reveal relationships among people instead of discovering associations among variables (16). It can provide an opportunity to uncover subgroups that have common neglect profiles and their correlates but with important distinctions. Therefore, we investigated whether older adult care workers can be divided into distinct subtypes based on their traits and behaviors using LPA, which can both provide a focused intervention for care workers to lessen the impact of neglect and help administrators of long-term care facilities in screening for appropriate older adult care workers during recruitment.

## Methods

### Sample and measures

Shandong University's Institutional Review Board approved the study protocol. In this study, the post-stratified convenience sampling method was used. Shandong Province was divided into five layers according to the administrative region, and one city was randomly selected from each layer. Among the registered long-term care facilities in each city, three facilities were conveniently sampled according to the different forms of operation (public operation, state-found-private-run, and private). Therefore, 15 institutions were selected from the total number of 962 institutions with complete medical and nursing certificates in Shandong Province (Data from Xinhua News Agency Jinan, 29 July 2023).

Data were collected through an online and offline questionnaire survey. The principal person in charge of each long-term care facility gave their consent before the survey was conducted. We selected the respondents based on the inclusion and exclusion criteria, and after explaining the research purpose, significance, and fulfilling requirements, we assured every respondent that the completed data would be kept anonymous and confidential. We obtained written informed consent from the participants. No privacy information was involved during the data collection process, and the ethical principles of voluntary participation, anonymity, and confidentiality were guaranteed. To avoid repeated filling of questionnaires, the same facility was surveyed using only one form of questionnaire (online or offline). Each question in the online questionnaire was set as a mandatory field and the offline questionnaire was collected face-to-face by the researcher to ensure that no questions were missed during the completion of the questionnaire. For those with low educational levels or dyslexia and who could not fill the questionnaires independently, the questionnaire items were read out to them and their responses were documented through face-to-face means. The care workers who could not understand the content of the questionnaire were excluded due to the possibility of serious personal subjectivity and bias. Among the 15 long-term care facilities, only two facilities opted for the online questionnaire due to the time conflict between the research schedule and the vacation of nursing staff in the institutions. The response rate of those who filled out the online questionnaire was 93.75%, and four questionnaires were eliminated because they were not carefully filled. The response rate of those who filled out the offline questionnaire was as high as 98.63%, and five questionnaires were not completed due to reluctance to fill the questionnaire and due to other reasons. A total of nine questionnaire responses were excluded. Two investigators independently reviewed the data.

The inclusion criteria were (1) older adult care workers (those who provided care to older adults aged 60 years and above); (2) care workers aged 18 years and above; (3) care workers who have worked in nursing institutions for 3 months and longer (17); and (4) care workers who provided informed consent to participate in the study. The exclusion criteria were older adult care workers (1) who could not understand the questionnaire after explanation and (2) who were on leave and who had left work for training and further studies.

### The Elder Neglect Scale for Geriatric Nursing Assistants (ENS for GNA)

The Elder Neglect Scale for Geriatric Nursing Assistants (ENS for GNA) (17) assessed older adult neglect. The scale includes 4 dimensions and 17 items: physical neglect (5 items), psychological neglect (4 items), economic neglect (3 items), and medical neglect (5 items). Each item was scored on a Likert scoring scale of 0–5, with total scores ranging from 0 to 85; higher scores indicate the lower risk of neglecting older adults. The rationality of the scale among the nursing population was verified. The scale has good credit validity, with a Cronbach's α coefficient of 0.877, a test-retest reliability of 0.944, and a content validity of 0.984. Zhang et al. (18) tested the reliability and validity of the scale in a study of older adult care workers, and the Cronbach's α coefficient was above 0.80.

### Chinese big five personality inventory brief version

The Chinese Big Five Personality Inventory brief version (19) contains 5 dimensions, each containing 8 items for a total of 40 items: neural, rigor, agreeableness, extraversion, and openness. The responses were graded using a 6-level Likert scoring scale, ranging from 1 to 6 points. The total score for each dimension was summed up, and it ranged from 8 to 48. The higher the dimension score, the more obvious the personality tendency. The Cronbach's α coefficient of all the five dimensions was above 0.75, with a minimum of 0.76 (agreeableness), a maximum of 0.81 (neuroticism), and an average of 0.79. We tested the reliability and validity of the scale in this study, and the Cronbach's α coefficient was 0.891.

### Demographic information

The research team designed the form based on the relevant previous literature to obtain demographic information. The following data were obtained: age, sex, marital status, education level, monthly income, employment qualification certificate, nature of the facility, regular training attendance, years of experience in the provision of older adult care, and number of older adults who are nursed simultaneously.

### Statistical analysis

We used LPA to identify traits of neglect in older adult care workers. Despite the possible arbitrariness of LPA in determining the number of class members due to its semi-subjective properties, its misclassification rate is relatively low, and it can produce more reasonable results compared with those of other classification approaches (20). We used Mplus v8.3 to identify the number of distinct care worker subtypes, the relative size of each subtype, and the distribution of characteristics within each subtype (15). Using stepwise addition, k+1 classes were added sequentially until the optimal solution for the data was obtained. The Akaike information criterion (AIC) is a standard for measuring the goodness of fit of statistical models. When choosing the best model from a set of models, the model with the smallest AIC is usually selected. The Bayesian information criterion (BIC) is similar to the AIC and is used for model selection. The BIC considers the number of samples. When the sample size is too large, the BIC can effectively prevent the model complexity caused by excessive model accuracy. The sample-size adjusted BIC (aBIC) is also used to assess the model fitting effect. Entropy is a measure from zero to one of how well individuals are assigned to latent classes (class differentiation). Entropy evaluates the classification quality of a model, with a value closer to 1 indicating a more accurate classification. The Lo–Mendell–Rubin likelihood ratio test and the bootstrap likelihood ratio test are used to examine the fit differences across different category models, demonstrating that when statistically significant, the k-category model beats the k-1 category model. When the category model for evaluating index tendency is inconsistent, the optimal model is chosen by completely measuring the outcomes of each index, considering their clinical relevance, and integrating the principles of interpretability and brevity.

After determining the best latent profile model and defining the classifications, statistical analysis was performed using SPSS25.0. Normally distributed continuous data were expressed as mean ± standard deviation, and categorical data were expressed as frequency and percentage (%). The Chi-square test or one-way analysis of variance was used to assess the correlation of demographic information and personality with different characteristics of neglect. Statistically significant variables in the univariate analysis were further included in a stepwise multivariate logistic regression analysis. The parallelism test (*x*^2^ = 40.096, *P* = 0.003) was significant, thus multivariate disordered logistic regression analysis was performed to evaluate the influencing factors of different latent categories of neglect characteristics. Odds ratios (ORs) and corresponding 95% confidence intervals (95% CIs) were calculated to assess the results of the regression analysis. The test level was α = 0.05, and *P* < 0.05 was considered statistically significant.

## Results

### Sample characteristics

In this study, 430 older adult care workers were selected from 15 long-term care facilities in Shandong Province from June to September 2022. Nine invalid questionnaires were excluded, and the final sample was 421. Older adult care workers were mainly young and middle-aged adults and mostly women (80.05%). Most of them were married (75.30%), 344 had the employment qualification certificate (81.71%), 293 attended training regularly (69.6%), and one-third provided care for more than four older adults simultaneously (35.34%). The monthly income of the participants was ¥3,001–5,000, accounting for 69.36% of the participants. The nature of long-term care facilities was mainly a public mode of operation, accounting for 58.67% of the included facilities. Additional information is presented in Table 1.

**Table 1:** The sample characteristics of older adult care workers (*N* = 421).

**Item**	**Classification**	**Number/ score**	**Percentage (%)**
Sex	Male	84	19.95
	Female	337	80.05
Age	20~40	189	44.89
	41~60	221	52.50
	>60	11	2.61
Marital status	Unmarried	72	17.10
	Married	317	75.30
	Other	32	7.60
Years of old care work	< Half a year	20	4.75
	Half a year ~1 year	72	17.10
	1 year~3 years	141	33.49
	3 year~5 years	69	16.39
	≥5 years	119	28.27
Education level	Illiteracy	21	4.99
	Elementary education	123	29.22
	Secondary education	269	63.89
	Higher education	8	1.90
Monthly income	< 2,000 yuan	19	4.51
	2,000~3,000 yuan	48	11.40
	3,001~4,000 yuan	136	32.30
	4,001~5,000 yuan	156	37.06
	>5,000 yuan	62	14.73
Nature of the facility	Public operation	247	58.67
	Private	88	20.90
	State-found-private-run	86	20.43
Have the employment qualification certificate	Yes	344	81.71
	No	77	18.29
Attend the training regularly	Yes	293	69.60
	No	128	30.40
Nursing number of older adults simultaneously	1~2	143	33.97
	3~4	129	30.64
	>4	149	35.39

### Latent profile analysis

The number of latent categories was gradually increased from one, and five latent category models were established. Table 2 displays the fitting indicators of the latent profile model of neglect among older adult care workers. The results show that AIC, BIC, and aBIC decreased with the number of latent categories, but the decline rate began to slow down when the number of latent categories was three. Entropy was maximum when the three categories were retained. Therefore, three-category models were developed as the optimal classification results of neglect among older adult care workers.

**Table 2:** The latent profile fitting indicator of neglect among older adult care workers (*N* = 421).

**Model**	**AIC**	**BIC**	**aBIC**	**Entropy**	**LMR (P)**	**BLRT (P)**	**Class probability**
1	8,705.067	8,737.408	8,712.022	—	—	—	100
2	8,068.494	8,121.048	8,079.795	0.910	0.0000	0.0000	0.68/0.32
3	7,953.258	8,026.025	7,968.905	0.931	0.0009	0.0000	0.53/0.34/0.13
4	7,877.995	7,970.976	7,897.989	0.901	0.0003	0.0000	0.51/0.19/0.19/0.11
5	7,822.934	7,936.128	7,847.275	0.899	0.0054	0.0000	0.44/0.19/0.18/0.11/0.08

The scores for each dimension of neglect in different latent profile categories are shown in Table 3. The subtypes of older adult care workers were assigned descriptive labels based on their characteristics/behaviors. Class 1: care workers in this group had the lowest score in each dimension (i.e., the degree of neglect) and a high risk of neglect of older adult care; they were labeled as the “high-risk group” (13.06%). Class 2: care workers in this group had their scores for each dimension between those of workers classified as C1 and C3 and were considered the “medium-risk group” (33.50%). Class 3: care workers in this group had the highest scores in all the dimensions (i.e., degree of neglect), and the older adults they cared for were the least overlooked; they were labeled as the “low-risk group” (53.44%).

**Table 3:** A comparison of scores for each dimension of neglect in different latent profile categories (*N* = 421).

**Category**	**Number**	**Physical neglect**	**Psychological neglect**	**Economic neglect**	**Medical neglect**
Low-risk group	225	24.15 ± 1.02	17.67 ± 2.36	12.27 ± 2.49	23.60 ± 2.07
Medium-risk group	141	19.59 ± 1.15	15.73 ± 2.38	10.41 ± 2.54	19.72 ± 2.75
High-risk group	55	14.69 ± 1.71	12.42 ± 2.89	8.24 ± 2.14	16.55 ± 2.30
*F* value		344.498	108.740	67.762	222.792
*P-*value		< 0.001	< 0.001	< 0.001	< 0.001

### Univariate analysis results

Univariate analysis was performed based on the LPA findings regarding the neglect behavior of older adult care workers. The results showed that factors such as sex; age; monthly income; nature of the facility; having an employment qualification certificate; attending training regularly; nursing a number of older adults simultaneously; and rigor, agreeableness, and extraversion personalities were statistically significantly different among the risk groups (*P* < 0.05; Table 4). However, no statistically significant difference in marital status and educational level was observed among the groups (*P* > 0.05). The high-risk group included the proportion of workers who were males, aged 20–40 years, with older adult care experience of <1 year, who were working in a private facility, with no employment qualification certificate, and who did not attend regular training was higher than that of those in the other two groups. The low-risk group included the proportion of workers who were female, with older adult care experience of more than 5 years, with a monthly income of more than ¥5,000, who were working in a public operation facility, with an employment qualification certificate, who regularly attended older adult care training, and who provided care for less number of older adults simultaneously was higher than that of those in the other two groups.

**Table 4:** Univariate analysis of neglect in different latent profile categories.

**Item**	**Classification**	**Latent profile categories**			***x*^2^/*F***	***P* value**
		**Low-risk group (225)**	**Medium-risk group (141)**	**High-risk group (55)**		
Sex	Male	31 (13.78)	26 (18.44)	27 (49.09)	*x*^2^ = 34.811	< 0.001
	Female	194 (86.22)	115 (81.56)	28 (50.91)		
Age	20~40	93 (41.33)	60 (42.55)	36 (65.45)	*x*^2^ = 15.605	0.048
	41~60	125 (55.56)	78 (55.32)	18 (32.73)		
	>60	7 (3.11)	3 (2.13)	1 (1.82)		
Marital status	Unmarried	39 (17.33)	22 (15.60)	11 (20.00)	*x*^2^ = 3.132	0.536
	Married	165 (73.33)	112 (79.43)	40 (72.73)		
	Other	21 (9.34)	7 (4.97)	4 (7.27)		
Years of old care work	< half a year	11 (4.89)	4 (2.84)	5 (9.09)	*x*^2^ = 16.512	0.036
	Half a year ~1 year	28 (12.44)	27 (19.15)	17 (30.91)		
	1 year~3 years	77 (34.22)	49 (34.75)	15 (27.27)		
	3 year~5 years	40 (17.78)	24 (17.02)	5 (9.09)		
	≥5 years	69 (30.67)	37 (26.24)	13 (23.64)		
Education level	Illiteracy	11 (4.89)	8 (5.67)	2 (3.64)	*x*^2^ = 5.292	0.726
	Elementary education	70 (31.11)	41 (29.08)	12 (21.82)		
	Secondary education	139 (61.78)	91 (64.54)	39 (70.90)		
	Higher education	5 (2.22)	1 (0.71)	2 (3.64)		
Monthly income	< 2,000 yuan	8 (3.56)	8 (5.67)	3 (5.45)	*x*^2^ = 22.756	0.004
	2,000~3,000 yuan	16 (7.11)	20 (14.18)	12 (21.82)		
	3,001~4,000 yuan	69 (30.67)	44 (31.21)	23 (41.82)		
	4,001~5,000 yuan	93 (41.33)	54 (38.30)	9 (27.85)		
	>5,000 yuan	39 (17.33)	15 (10.64)	8 (15.36)		
Nature of the facility	Public operation	153 (68.00)	72 (51.06)	22 (40.00)	*x*^2^ = 21.429	< 0.001
	Private	32 (14.22)	37 (26.24)	19 (34.55)		
	State-found-private-run	40 (17.78)	32 (22.70)	14 (25.45)		
Have the employment qualification certificate	Yes	197 (87.56)	118 (83.69)	29 (52.73)	*x*^2^ = 36.428	< 0.001
	No	28 (12.44)	23 (16.31)	26 (47.27)		
Attend the training regularly	Yes	178 (79.11)	88 (62.41)	27 (49.09)	*x*^2^ = 23.996	< 0.001
	No	47 (20.89)	53 (37.59)	28 (50.90)		
Nursing number of older adults simultaneously	1~2	92 (40.89)	44 (31.21)	7 (12.73)	*x*^2^ = 25.734	< 0.001
	3~4	71 (31.56)	33 (23.40)	25 (45.45)		
	>4	62 (27.55)	64 (45.39)	23 (41.82)		
Big five personality	Neural	22.56 ± 10.53	22.51 ± 10.47	22.47 ± 10.53	*F* = 2.116	0.122
	Rigor	36.61 ± 7.49	36.61 ± 7.47	36.64 ± 7.51	*F* = 13.417	< 0.001
	Agreeableness	32.39 ± 6.04	32.36 ± 5.99	32.43 ± 5.99	*F* = 3.792	0.023
	Extraversion	33.64 ± 8.11	33.62 ± 8.10	33.59 ± 8.16	*F =* 4.630	0.010
	Openness	30.53 ± 6.96	30.49 ± 6.94	30.53 ± 6.96	*F =* 1.925	0.147

### Multivariate logistic regression analysis results

Variables with statistical significance in the univariate analysis were included as independent variables and covariates, and three potential categories, namely, high-, medium-, and low-risk groups were analyzed as dependent variables. The results showed that males in the high-risk group had a 3.293 times higher risk of neglect than that observed in the low-risk group (OR = 3.293, *P* = 0.006). Older adult care workers with low monthly income in the medium-risk group had a 3.034 times higher risk of neglect than that observed in the low-risk group (OR = 3.034, *P* = 0.002). Older adult care workers with an employment qualification certificate (OR = 0.361, *P* = 0.018), who attend training regularly (OR = 0.572, *P* = 0.035), and who nurse <4 older adults simultaneously (OR = 0.474, *P* = 0.012) were less likely to exhibit neglect behaviors, as shown in Table 5.

**Table 5:** The results of multivariate logistic regression analysis of neglect potential profiles.

**Item**	**Medium-risk group vs. Low-risk group**				**High-risk group vs. Low-risk group**			
	β	***OR*** **(95%*****CI*****)**	***Wald*** *x*^2^	***P***	β	***OR*** **(95%*****CI*****)**	***Wald*** *x*^2^	***P***
Sex	0.100	1.106 (0.572~2.136)	0.089	0.765	1.192	3.293 (1.405~7.721)	7.519	0.006
Monthly income	1.138	3.121 (1.160~8.397)	5.080	0.024	0.424	1.527 (0.394~5.916)	0.376	0.540
Have the employment qualification certificate	−0.048	0.953 (0.478~1.899)	0.019	0.890	−1.02	0.361 (0.155~0.839)	5.598	0.018
Attend the training regularly	−0.558	0.572 (0.341~0.961)	4.450	0.035	−0.461	0.631 (0.292~1.361)	1.381	0.240
Nursing number of older adults simultaneously	−0.746	0.474 (0.264~0.851)	6.265	0.012	−1.068	0.344 (0.117~1.013)	3.749	0.053

## Discussion

### Overall findings

This study identified three potential categories of older adult care workers, namely, high-, medium-, and low-risk groups of neglect, using LPA. Nearly half of the older adult care workers were at high or medium risk of exhibiting neglect behaviors. Additionally, this study considered the traits and motivating elements of these groups, which is anticipated to aid the management of long-term care facilities in identifying caregivers who are at high risk of exhibiting neglect behaviors and in developing intervention plans.

The results showed that the incidence of neglect of care was lower in this study than in the study by Zhang et al. (18). Although both studies are on older adult care workers in long-term care facilities, more than half of the respondents in Zhang et al.'s study worked in private facilities. The funds and resources available for private facilities are less abundant than those of public-operated facilities. These private facilities impose a heavy burden on care workers, have poor professional nursing staff, and have a weak ability to provide high-level nursing service for older adults, which can likely to lead to a higher neglect rate. The following were identified as the causes of the lower rate of neglect in this study: (1) long-term care facilities focus on the skill training and service quality of older adult care workers to attract more older adults; this ensures that the older adults receive improved care; (2) the majority of older adult care workers who completed the questionnaire provided care to only few older adults who were bedridden, indicating that the older adults had sufficient self-care abilities and did not require as much assistance from older adult care workers (21). Although the possibility of neglect was low in this study, it cannot be treated lightly in future studies. The training of older adult care workers should be strengthened to help them better understand the needs of older adults and reduce the occurrence of neglect.

### Factors influencing neglect

The results of this study showed that the proportion of males was the largest in the high-risk group, which is consistent with previous studies (22). Alon et al.'s (23) research showed that three-quarters of the abusers were men. Arens et al. (24) also reported that men compared with women were more likely to neglect care. This finding may be related to the personalities. Men are generally not careful, while women are more meticulous, more thoughtful, and more patient with their work and lifestyle compared with that exhibited by men. Thus, women provide more comprehensive care to older adults and do not easily neglect the provision of care.

This study showed that not having an employment qualification certificate can easily lead to the neglect of care, which is consistent with Zhang et al.'s findings (18). Identifying neglect in daily life is difficult. Due to a lack of professionalism, older adult care workers without employment certificates may be unable to identify abuse or neglect events. Jiao et al.'s (25) research showed that older adult care workers with employment certificates scored higher in older adult care knowledge, interpersonal communication, psychological nursing, professional nursing knowledge, professional ethics, service etiquette, and emergency skills than the scores of non-certificated care workers, with statistical significance.

Older adult care workers who attend training regularly are less likely to neglect care. Those who failed to attend related training due to short rest time or low enthusiasm for related training did not improve their knowledge of older adult care at work. This results in inadequate knowledge and skills on older adult care and the inability to meet the needs of older adults (26), as well as increasing the probability of the occurrence of neglect.

In the medium-risk group, care workers with low monthly income were three times more likely to neglect care than those observed in the low-risk group, and the lower the income, the greater the probability of neglect. Liu et al. (27) found that 62.9% of older adult care workers had a low income, which was similar to the findings by Wan et al. (28). Their study showed that most older adult care institutions generally have problems such as the mismatch between salary level and labor intensity and poor treatment and welfare, and some institutions do not pay social insurance for older adult care workers (29). Income is the manifestation of a person's return for labor. In cases of the low-income level and high labor intensity, older adult care workers feel insecure. They assume that the job is extremely unstable to sustain passion over a long time, so they eventually neglect the provision of care to older adults.

The results showed that the low-risk group had care workers attending to fewer older adults simultaneously, which is similar to the findings of previous studies (30). In contrast, if the number of older adults is >4, care workers can only prioritize to meet the basic living needs of these older adults within their ability. These workers will ignore or cannot participate in psychological, rehabilitation, or medical projects (31).

The logistic regression results showed no statistically significant difference between personality characteristics and neglect. This is different from our previous assumption. Each kind of work has different requirements regarding work skills, psychological quality, and personality traits, and workers' own stable personality characteristics can provide an important reference for care facilities in the hiring and promotion of suitable candidates, matching individuals with specific work (32).

### Implications for long-term care facilities

The results of this study showed that the proportion of the high-risk group was 13.06%, and attention should be paid to older adult care workers in this group. These workers had low scores in all dimensions of neglect. As more older adults choose to remain in long-term care facilities, an imbalance between the number of care workers and allocated tasks may be observed. The reasons for psychological neglect among older adults may stem from a lack of professional skills and knowledge in psychological nursing, as well as the heavy daily work burden. Consequently, older adult care workers may inadvertently pay less attention to the psychological wellbeing of older adults. The cause of economic neglect may be due to the special nature of “money” and property, which are more private and sensitive issues for most older adults. If care workers manage older adults' money, some financial disputes could occur; therefore, they deliberately avoid involving themselves in the financial events of older adults (18). The cause of medical neglect may be due to the presence of some professional gap between medical staff and caregivers. For example, bedridden older adults do not feel convenient to express their feelings, and care workers can consequently ignore their poor health status (18). Therefore, care workers should pay more attention to the psychological condition of older adults, improve the attention given to the properties of older adults, timely observe and report the physical health of older adults, fundamentally reduce the occurrence of neglect, and improve the quality of life of older adults.

Care workers have a responsibility to ensure the safety of older adults who rely on them, protect them from abuse and neglect, and also ensure that older adults do not pose a risk to their families and any visitors they may have. The case described by Corbi clearly illustrates that neglecting the basic emotional needs of an older adult may lead to sexual abuse of others (33). Therefore, while taking care of older adults, more attention should be paid to their emotional needs. Long-term care facilities should establish mental health counselors to provide psychological counseling and emotional communication for older adults and strengthen the supervision of potential abusers.

This study found that males posed a high risk of neglect of care in long-term care facilities. This suggests that facilities should constantly improve the skills of male older adult care workers through lectures and attending education abroad to reduce the occurrence of neglect. Few studies have been performed on the effect of sex on neglect among older adult care workers in China, and further follow-up studies are needed to verify the effect of sex on neglect.

Identifying neglect in daily life is difficult. In some instances, neglect of care would have occurred, but the care workers were unaware of its occurrence, and in other instances, the care workers might not know what abuse or neglect is. The results of Corbi's survey also demonstrate that the level of awareness and perception of older adult abuse by healthcare professionals are still very poor, especially regarding the manner of reporting (34). The majority of nurses and the care assistants declared that they never had suspicions of abuse, and 50% of the nurses and 62.5% of the care assistants were unaware of standard reporting procedures. Meanwhile, nurses and physicians also lack knowledge about older adult abuse issues and the related laws. This phenomenon reflects the importance of the employment qualification certificate. In this study, although only a few care workers did not have employment qualification certificates, most of them did not pay attention to abuse and neglect as a problem in the provision of older adult care. Furthermore, the poor attitude of some practitioners toward older adults and the inability to relieve the work pressure lead to neglect. This suggests that older adult care workers should be actively encouraged to participate in the grade examination for obtaining the employment qualification certificate. In addition, the topic of older adults abuse and neglect education and specific assessment should be included in the training course. The relevant concepts and scope of abuse and neglect of older adults should be clearly defined, and the importance of older adult abuse in society and law should be emphasized. In-depth training and education should be provided to older adult care workers from both theoretical and real-life cases to facilitate timely detection and reporting of potential older adult abuse incidents and to reduce the occurrence of neglect.

The enthusiasm of older adult care workers toward participating in regular professional training should be improved, additional time should be provided, and older adult care workers should improve their energy for studies. The training courses should be scientifically and reasonably planned and fully combined with the actual training needs of the older adult care workers. More flexible methods, such as training methods for flipped classrooms or simulation model drills (35), can be adopted during the training while ensuring the participation of older adult care staff.

The low-income level is the main cause of neglect. The government can formulate relevant subsidy policies, fully implement the salary payment and subsidies for older adult care workers in long-term care facilities, and actively advocate for these institutions to pay their social insurance. Long-term care facilities should actively implement incentive policies to provide appropriate rewards for the spiritual, material, or economic aspects of care provision to maintain the enthusiasm of care workers and reduce the occurrence of neglect.

This study found that a large gap in the number of care workers and high labor intensity still exists, resulting in the increasing burden and risk of neglect. The government should actively lead the development of undertakings for older adult care workers, relieve the work pressure of older adult care workers by recruiting part-time nurses and experienced social workers, and support the training of these workers at an early stage. Long-term care facilities should reasonably schedule the amount of care and attempt to balance the supply-demand ratio. Colleges and universities should support the training of nurses interested in older adult care, promote employment agreements between colleges and long-term care institutions, and address the problem of proportion imbalance during the training of nurses.

This study did not conclude on a difference between personality traits and neglect. Future research can further explore the detailed relationship between personality characteristics and neglect through other approaches, such as the Traditional Chinese Medicine Constitution. This will help managers schedule workers for the most appropriate positions according to their different personalities and specific work requirements, which will ensure that they can make the best use of their talents (36).

## Limitations and future directions

This study innovatively divides older adult neglect into different categories based on LPA for the first time and explores the influencing factors of neglect, providing an empirical study on the problem of neglect in the older adult care service system. This study has some limitations. First, this study only analyzed the differences in demographic data and personality characteristics of different categories of care workers, which may have other factors. Our study used a post-stratified convenience sampling method, which may have led to potential selection bias. In addition, this study was conducted in only Shandong Province, and the scope of sample collection was limited, which can limit the generalizability of the findings. Geographic and cultural differences may lead to a lack of representativeness in the results. Therefore, caution is needed when applying these findings to other regions or populations. In the future, other variables can be added to investigate large sample studies in different regions. Second, we used a cross-sectional design with no longitudinal follow-up of neglect. Our data only presents a snapshot of the current situation and does not permit causal inferences. Longitudinal studies should be conducted in the future to analyze influencing factors more accurately.

The sensitive nature of older adult neglect is also worth mentioning. Although before the beginning of the survey the researchers informed the older adult care workers of the research purpose and assured them of confidentiality, some workers may still be worried that the survey content will affect their work. This may result in untruthful answers due to concerns or fear, which can cause different degrees of information bias and subsequently influence the accuracy of the conclusion. Future studies should access information through multiple approaches, such as increasing objective assessments of older adult care or obtaining information from managers and older adults to ensure the reliability of the data.

This study found heterogeneity in the neglect of older adult care workers and divided the workers into three potential categories based on the LPA method. The study explored neglect of older people, complemented the existing literature, and provided directions for future research. Male older adult care workers and workers with low monthly income had a higher risk of neglect. Older adult care workers with employment qualifications, who attended training regularly, and who provided care to < 4 older adults simultaneously had less risk of neglect. The managers of long-term care facilities should understand the individual characteristics of employed older adult care workers and identify and focus on the high-risk group of older adult care workers. In the future, different categories of influencing factors can be considered as the focus of intervention measures to reduce the occurrence of problems due to neglect, provide scientific guidance, and help long-term care facilities to develop policies regarding older adult care.

## Data availability statement

The raw data supporting the conclusions of this article will be made available by the authors, without undue reservation.

## Ethics statement

The studies involving humans were approved by Shandong University's Institutional Review Board, Shandong University. The studies were conducted in accordance with the local legislation and institutional requirements. Written informed consent for participation in this study was provided by the participants' legal guardians/next of kin.

## Author contributions

CY: Data curation, Writing – original draft, Conceptualization. CW: Writing – original draft. XD: Writing – review & editing. LS: Writing – original draft. WG: Writing – review & editing. DL: Supervision, Writing – review & editing.
