# Supplementary material for: Profiles in neglect of older adult care workers in a long-term care facility: a latent profile analysis
Source: Front Public Health. 2024 Mar 25;12:1320896. doi: 10.3389/fpubh.2024.1320896 (PMC10999726; doi:10.3389/fpubh.2024.1320896)
Supplement: Supplementary file 1 [file Data_Sheet_1.PDF]

Sociodemographic questionnaires

1.Your gender ☐male ☐female

2.Your age ______

3.Your Education level ☐illiteracy ☐elementary education ☐secondary education ☐higher education

4.Your marital status ☐unmarried ☐married ☐other

5.Your monthly income ☐<2000yuan ☐2000~3000yuan ☐3001~4000yuan ☐4001~5000yuan ☐＞5000yuan

6.Your years of old care work ☐half a year ~1 year ☐1 year~3 years ☐3 year~5 years ☐≥5 years

7.Your nature of the facility ☐ public operation ☐ private-private ☐ state-found-private-run

8.Do you have the employment qualification certificate ☐Yes ☐No

9. Do you attend the training regularly ☐Yes ☐No

10. Nursing number of older adult simultaneously ☐ 1~2 ☐ 3~4 ☐ ＞4

Chinese Big Five Personality Inventory brief version

Please tick "√" below that suits your situation	Not at all	Most don't	Kind of non-fits	Kind of fits	Most do	Exactly
1. I'm always worried that something bad is going to happen	1	2	3	4	5	6
2. I was always scared	1	2	3	4	5	6
3. Sometimes I feel like I'm worthless	1	2	3	4	5	6
4. I rarely feel depressed *	1	2	3	4	5	6
5. A casual remark from someone else, I often associate it with myself	1	2	3	4	5	6
6. I felt like I was about to collapse when I was under pressure	1	2	3	4	5	6
7. I often worry about things that don't matter	1	2	3	4	5	6
8. I often feel unsettled	1	2	3	4	5	6
9. At work, I always just want to be able to cope with the past*	1	2	3	4	5	6
10. Once I have set a goal, I will continue to work hard to achieve it	1	2	3	4	5	6
11. I often make decisions after careful consideration	1	2	3	4	5	6
12. People think I'm a cautious person	1	2	3	4	5	6
13. Logic and organization are a hallmark of mine.	1	2	3	4	5	6
14. I like to plan things out right from the start.	1	2	3	4	5	6

15. I work or study hard	1	2	3	4	5	6
16. I'm a person who does everything I can	1	2	3	4	5	6
17. Despite the darker aspects of human society, I still believe that human nature is generally good	1	2	3	4	5	6
18. I think most people are basically well-intentioned	1	2	3	4	5	6
19. Although there are some liars in society, I think most people are still credible	1	2	3	4	5	6
20. I don't really care if people are treated unfairly*	1	2	3	4	5	6
21. I often feel that other people's pain is none of my business.	1	2	3	4	5	6
22. I often feel sorry for those who have suffered.	1	2	3	4	5	6
23. I'm the kind of person who only takes care of myself and doesn't worry about others*	1	2	3	4	5	6
24. I often feel sad when people tell me about their misfortunes.	1	2	3	4	5	6
25. My imagination is quite rich	1	2	3	4	5	6
26. My mind is always full of vivid images	1	2	3	4	5	6
27. I have a strong curiosity about many things	1	2	3	4	5	6
28. I love adventure	1	2	3	4	5	6
29. I'm a risk-taking, out-of-the-box person	1	2	3	4	5	6
30. I have an adventurous spirit that no one else has	1	2	3	4	5	6

31. I'm eager to learn new things, even if they have nothing to do with my daily life	1	2	3	4	5	6
32. I am willing and easy to accept new things, new perspectives, new ideas	1	2	3	4	5	6
33. I love to socialize and have fun	1	2	3	4	5	6
34. I'm bored with crowded parties*	1	2	3	4	5	6
35. I try to avoid crowded parties and noisy environments*	1	2	3	4	5	6
36. At lively parties, I often take the initiative and have fun	1	2	3	4	5	6
37. When I'm around, it's usually a good time	1	2	3	4	5	6
38. I want to be a leader rather than be led	1	2	3	4	5	6
39. In a group, I want to be in a leadership position	1	2	3	4	5	6
40. People think of me as a warm and friendly person	1	2	3	4	5	6

Elder Neglect Scale for Geriatric Nursing Assistants

Please tick "√" below that suits your situation	Never/Not applicable	Rarely	seldom	sometimes	often	Always
1. Do you clean the rooms and beds where the elderly live every day?	0	1	2	3	4	5
2. Do you recognize these needs of the elderly and give assistance, such as bathing/dressing/undressing/feeding/toileting/assisting/combing hair/trimming nails/cleaning mouth？	0	1	2	3	4	5
3. Do you pay attention to the needs of the elderly to communicate with family, friends or other people who live with them？	0	1	2	3	4	5
4. Would you pull up the bed stop for the bedridden elderly？	0	1	2	3	4	5
5. Do you know the physical condition of the elderly and the diseases they are suffering from？	0	1	2	3	4	5
6. Will you accompany the elderly to chat and walk？	0	1	2	3	4	5
7. Do you contact the doctor in time when the elderly are sick？	0	1	2	3	4	5
8. If an elderly person has lost or damaged items or belongings, will you deal with it promptly？	0	1	2	3	4	5
9. Do you remind or help the elderly to wear clothing that is appropriate for the	0	1	2	3	4	5

weather and season？						
10. Do you understand that the elderly have discretionary possessions？	0	1	2	3	4	5
11. Have you ever taken medication inappropriately (failed to take medication on time/overdosed/underdosed/missed dose) in the process of taking care of the elderly？	0	1	2	3	4	5
12. Do you understand the consumption needs of the elderly, such as buying clothes and daily necessities？	0	1	2	3	4	5
13. Do you close the bed curtain or cover it with an object when you handle urine and urination for the elderly？	0	1	2	3	4	5
14. The elderly take such as antihypertensive drugs, oral hypoglycemic drugs or insulin injection when the adverse reaction, you will promptly contact the doctor？	0	1	2	3	4	5
15. Will you comfort and enlighten the elderly who are in a bad mood？	0	1	2	3	4	5
16. Do you sometimes forget to carry out a treatment or care plan because you are so busy with work？	0	1	2	3	4	5
17. Do you turn over an elderly person who has been bedridden for a long time every two hours？	0	1	2	3	4	5
